# Notes and News

**Published:** 1892-07-30

**Authors:** 


### Notes and News.

Collected and Communicated.

A correspondent to the *Globe* makes a suggestion in connection with Hospital Saturday Street Collections, which is worthy of consideration. He proposes that contributors should be provided with a ticket notifying that they have contributed, which should prevent them being subjected to further solicitations.

We trust that the Italian Hospital will have benefited to a large extent from the entertainment given by Mr. Imré Kiralfy at Olympia last week. The entertainment was under most distinguished patronage, the King of Italy acting as patron. After the spectacle a battle of flowers took place between the occupants of gondolas, which were charmingly decorated with flowers.

A New Orleans paper, in an article entitled "Too Much Charity," reveals to us the enormous sums which find their way to the charity coffers of America. It is stated that legacies to charitable institutions exceed those of any other country. The New York State Board of Charities reports that during the past year property devoted to charitable purposes amounted to 54,000,000 dols. This does not include the money raised by small societies and private persons. It is stated that 64,322 in-patients were treated in the hospitals and 385,622 out-patients, and 48,500 persons were assisted with money. That is, 498,894 persons, or one-twelfth of the entire population of the State, received charity or public aid of some kind. It is calculated that 600,000 people in New York receive each year [illegible]30,000 dols. in charity. The writer of the article we quote from continues: "One tenth of the population of the State are more or less paupers, and the charity tax is larger than any other." "We were wont to boast that no man need be a pauper in the United States, that there is always a chance of making a living here, and that we would never be as bad as England in this respect; but we are rapidly drifting in that direction, and the army of those who call for public aid is steadily growing larger Let us hope that charity, without refusing aid wherever it is really needed, will be more carefully given, and that it will not help to develop a dangerous pauperism."

The new temporary sanatorium at Cardiff is now in working order. It is situated in an open field. The two wards are light and airy, and very complete in arrangement. It is intended that the building shall be utilised as a small-pox hospital, as soon as the permanent sanatorium in course of erection on Ely Common is completed.

The Chairman of the Great Northern Central Hospital makes a special appeal to the public to assist him in obtaining the fulfilment of a promise on the part of a friend to present the hospital with £500, provided nine other persons can be found to subscribe a like sum. Increased accommodation is greatly required at the institution, the present number of beds having been found to be entirely inadequate.

There is a society in our midst concerning which comparatively few of us are acquainted. This society is the Metropolitan Provident Medical Association. It may be considered a Medical Benefit Society, co-operating with local doctors and the hospitals. Branches of the work now number sixteen, of which twelve are dispensaries and four medical clubs. New branches are contemplated in Poplar, Woolwich, and Willesden. Seventy medical men, eleven dentists, and ten midwives carry on the work of the association, and have under their care over 25,000 persons. This number is likely to increase rapidly, as many working men's clubs, benefit societies, and temperance organizations desire to affiliate with the association. A further development will be in the direction of a supply of trained nurses to attend members when required. This will add value to the work of a society increasingly appreciated by the working classes.

The friends of the Gordon Boys' Home have made strenuous efforts to place this memorial scheme on a sound financial basis, but a very much larger response to their appeals must be made, than has hitherto been the case, before the Home can be regarded independent of the large donations it has received in the past. The work is of national importance, for those with whom it is concerned would probably tend to swell the large class of "ne'er do wells," instead of becoming useful members of the community. A visit to the Home at Cobham convinces one alike of the difficulty of the task undertaken, and of the very creditable result attained. Most of the inmates have been under no sort of discipline before entering, and as they are not received under fourteen years of age, the habits they have acquired are eradicated with difficulty. Nevertheless, the tabulated report of the Home shows that 55 per cent. of the boys have received very good reports on leaving, 33 per cent. have been reported as good, whilst only 12 per cent. have been indifferent or bad. During their residence in the Home, such mental education as is required in each individual case is given, and all are taught a trade, selected by themselves. Sir Dighton Probyn is endeavouring to accumulate a fund to be invested to endow the Home, and to this the Princess of Wales has contributed £500. Were all as fully acquainted as the Princess with the value of such an undertaking to the whole nation, we feel sure the £40,000 appealed for would be forthcoming.

